# Social expectations or self-regulation?—study on tourists' comity behavior in taking photos

**DOI:** 10.3389/fpsyg.2025.1492283

**Published:** 2025-05-27

**Authors:** Dong Li, Zhaowen Duan, Xiaoliang Xu, Ying Chen, Xuyi Liu

**Affiliations:** ^1^School of Tourism, Xi'an International Studies University, Xi'an, China; ^2^Shanghai Cooperation Organization National Tourism Cooperation and Cultural Exchange Research Center, Xi'an International Studies University, Xi'an, China; ^3^School of Tourism, Xinjiang University of Finance and Economics, Ürümqi, Xinjiang Uyghur Region, China

**Keywords:** social norms, individual norms, courteous behavior in photography, impression management motivation, proactive personality

## Abstract

**Introduction:**

During travel, photos capture beautiful moments and serve as a visual narrative. However, achieving a satisfactory photo in crowded tourist scenes relies on tourists' mutual cooperation and courtesy. This study aims to explore tourists' courteous behavior in photo-taking through normative activation theory.

**Methods:**

Structural equation modeling.

**Results:**

(1) Both social and individual norms positively affect courteous behavior, with individual norms having a greater impact; (2) Impression management motivation mediates the relationship between norms and courtesy; (3) Proactive personality moderates the link between individual norms and impression management but does not affect the link between social norms and impression management.

**Discussion:**

This study expands the application of normative activation and impression management theories and offers practical insights for tourism management to encourage civilized behavior.

## Introduction

Photographing, as an art form that captures moments, endows travelers with the ability to visually narrate stories and immortalize the natural beauty and emotional experiences encountered during their journeys. Photos serve not only as a means for tourists to treasure beautiful moments but also as a bridge that connects personal experiences with social interactions (Li et al., 2025). However, the rise of mass tourism has led to crowded travel scenes and tourists flocking to take photos, causing resource strain and time competition. This situation has resulted in a series of uncivilized behaviors, such as disregarding queue order, occupying photo spaces excessively, blocking others' lines of sight, and intruding into others' photos. These behaviors not only impact the travel experience but also contribute to unnecessary friction and conflicts, affecting the emotions and experiences of tourists. While studies have explored the dimensions of civilized tourism (Li, 2016), theoretical frameworks (Zhou, 2021), driving mechanisms (Liu et al., 2024), behavioral intentions (Zhou et al., 2021), and the formation and evolution of uncivilized tourism behaviors (Wang et al., 2022), as well as their generative factors (Lu et al., 2019) and governance (Lin, 2024), an in-depth investigation into the formation mechanism of considerate photo-taking behavior in tourism contexts has not been conducted.

Tourist photo-taking etiquette behavior research integrates norm activation theory, impression management theory from social psychology, incentive and constraint mechanisms from behavioral economics, and moral judgment and behavioral norms from ethics. The intersection and integration of these theories provide a multidimensional analytical perspective to fully understand the occurrence mechanism and behavioral decision-making of tourist photo-taking etiquette behavior. Recently, scholars have theoretically discussed and empirically analyzed tourists' civilized and uncivilized behaviors from various perspectives. In unfamiliar travel environments, tourists may relax self-behavior constraints due to anonymity and unique circumstances, leading to normative deviation behaviors (Zhang, 2008; Liao and He, 2018). From the tourists' perspective, civilized etiquette behavior correlates closely with moral norms, personality traits, and self-management abilities (Qiu, 2016; Li and Bai, 2018). Conversely, uncivilized tourism behavior often stems from a weak sense of public order (Xiao, 2007), lack of courtesy in public spaces, strong competition, and disregard for rules (Hu, 2016). Despite extensive exploration of factors influencing civilized and uncivilized tourism behaviors, research on the formation mechanism of common tourist photo-taking etiquette behavior remains inadequate. Existing studies primarily focus on factors influencing these behaviors but neglect the formation mechanism of tourists' civilized etiquette behavior in detail. Furthermore, research tends to categorize tourism behavior without exploring the transmission mechanism between tourists' “norm activation” and “photo-taking etiquette behavior” in public places, limiting behavior predictability to some extent.

The present study adopts a multidisciplinary and interdisciplinary methodological approach, integrating theories from social psychology, behavioral economics, environmental psychology, and other fields to explore tourists' photo-taking etiquette behavior. This study aims to address the following questions: Does norm activation directly impact tourists' photo-taking etiquette behavior? Does motivation for impression management mediate the relationship between norm activation and photo-taking etiquette behavior? Is there a moderating effect of proactive personality on the relationship between norm activation and impression management motivation? This paper will develop an integrated model incorporating social norms, individual norms, impression management motivation, proactive personality, and photo-taking etiquette behavior, with tourists as the research subjects. Using structural equation modeling and combining theoretical frameworks with empirical testing, this study seeks to investigate the aforementioned research questions. It is hoped that this research will contribute to enriching the study of tourist behavioral norms and tourism ethics, providing both theoretical support and practical guidance for tourism management practices.

## Literature review

### Norm activation model

The Norm Activation Model is a theory proposed by Schwartz (1977) to explain and predict the specific behavior of individuals. Social norms refer to the perceived social pressures in individual behavior decision-making (Ajzen, 1991). A distinct demarcation exists between social norms and individual norms in the norm activation model. Social norms refer to the perceived social pressure individuals encounter during behavioral decision-making, primarily shaped by collective expectations and external pressures (Ajzen, 1991). Specifically, social norms predominantly encompass descriptive norms and injunctive norms (Cialdini et al., 1991). Descriptive norms are behavioral standards formed spontaneously by most people after assessing their situation and deeming certain behaviors correct (Zheng et al., 1997). Visitors often adhere to descriptive norms to adapt to their surroundings or to fit in with others. Prescriptive norms refer to systems, standards, and rules established by society or various organizations (Chen and Xie, 2018), reinforcing certain behaviors by recognizing accepted actions and punishing those that are not (Schütze et al., 2025). In the context of tourism, directive norms refer to the requirements and expectations set by destination managers and service personnel for tourists, which can take the form of signage or verbal instructions. These norms clarify the expectations for tourists' photo-taking behavior, providing direct reminders and normative constraints.

In contrast to social norms, individual norms stem from internalized values and moral convictions. These norms constitute an individual's cognitive framework for understanding personal responsibilities and obligations, representing an internalized sense of duty that reflects personal values. Scholars have extensively used this conceptualization to explain the fundamental drivers of altruistic behaviors (Li et al., 2022). Individual norms are direct factors driving individuals to take or give way in photos, influenced by the awareness of consequences and the attribution of responsibility. Specifically, in the context of photo-taking etiquette, tourists' individual norms involve viewing courteous photo-taking as part of their moral obligations, with failure to adhere leading to feelings of guilt.

Result consciousness generally refers to an individual's recognition of the negative outcomes from not engaging in a certain behavior. For photo-taking etiquette, it pertains to tourists' awareness of the negative consequences of uncivilized behaviors such as crowding, disorder, and selfish actions. The greater the perception of these adverse consequences, the higher the likelihood of engaging in courteous behavior. Attribution of responsibility involves the belief that one is responsible for the negative outcomes of not performing a certain act. In terms of photo-taking etiquette, it means tourists recognize their responsibility for the negative consequences of not practicing courtesy, such as photo congestion, prolonged occupation of public spaces, and hindering other tourists' ability to take pictures.

### Theory of impression management

The Theory of Impression Management was proposed by American sociologist and psychologist Erving Goffman (1959) in his book “The Presentation of Self in Everyday Life.” Impression management theory explains the interaction communication process of individuals and describes the behavioral characteristics of humans in social interactions. Goffman likens impression management to a drama, suggesting that one party in an interaction aims to control the behavior of others, eliciting a response that aligns with their own intentions through their behavior.

During travel activities, especially when accompanied by family, relatives, friends, or colleagues, tourists not only pay attention to their public image but also to the impressions of their travel companions, which can trigger more positive behaviors (Zhu and Xu, 2021). According to Baumeister (1982), impression management is the use of behavior to communicate information about oneself and others, with the aim of establishing, maintaining, or refining one's image in the minds of others. He shifted impression management from a conceptual curiosity to a scientific analysis of it as a fundamental interpersonal process. Similarly, Tetlock and Manstead (1985) argue that impression management involves “the use of strategies that create a favorable social image or social identity.” Both concepts reflect that impression management is related to maintaining an identity. Arkin (1981), starting with the definition of a situation, argues that impression management refers to the process and way in which an individual plans, adopts, and executes a self-image in a situation where they interact with others. Impression management theory explains the process by which people manage and control the impressions others form about them, attempting to influence others' perceptions and behaviors by controlling the information they receive (Mehra et al., 2023; Vaghefi et al., 2024). Although most tourists are strangers in the tourism context, they strive to make a good impression and be viewed positively, driven by impression management motivation (Rosenfeld et al., 1995). While impression management often inspires positive behaviors, it must be acknowledged that people sometimes take unwanted actions to make a good impression on others (Li and Bai, 2018).

It is evident that to improve their self-image, tourists will adopt relevant behaviors based on impression management motivation, whether actively or passively (Yang and Zhang, 2022). In tourism activities, tourists leave their usual environment, which may reduce the motivation for impression management, leading to inappropriate “letting go” behaviors. However, with the influence of social norms and self-regulation, impression management motivation can have a strong predictive effect on courteous behavior among tourists. Therefore, this study chooses impression management motivation as a mediating factor between norm activation and photo-taking etiquette behavior.

### Comity

In traditional Chinese ethical culture, comity is not merely an attitude but also a behavioral relinquishment of interests. Comity stands in direct contrast to contention—where contention disrupts harmony, comity precisely sustains it (Liu, 2021). Domestic research on comity predominantly focuses on ancient texts and employs speculative discourse. As a traditional virtue of the Chinese nation, comity has always held significant importance in social life. It is an indispensable part of daily human interactions and a cornerstone for building a harmonious society. During tourism activities, visitors naturally desire to fully appreciate scenic views, relax, and capture memorable moments. However, when certain tourists occupy prime photography positions, block pathways, or monopolize observation decks for extended periods, others' photographic needs are severely impacted. Such prolonged waiting and crowding not only cause dissatisfaction but may also provoke disputes, ultimately degrading the tourism experience. Integrating the essence of comity with civilized tourism principles, this study defines tourists' comity behavior in photography contexts as the voluntary relinquishment of personal interests during photographic activities to accommodate others' needs.

The influence of traditional Chinese comity norms on individual norms is profound, shaping not only behavioral patterns but also contributing to the formation of social norms. This impact manifests particularly in tourism photography contexts. For instance, the cultural principle of “others before self” motivates tourists to prioritize others' photographic needs through proactive yielding, fostering a positive collective behavioral climate. Concurrently, traditional comity norms demonstrate intrinsic connections with modern norm activation theory. As norm activation theory emphasizes the formation and activation of individual norms, the moral tenets and values embedded in traditional comity norms serve as critical catalysts for activating tourists' behavioral standards. This mechanism drives tourists to consciously observe comity practices during photographic activities. Furthermore, traditional comity norms exhibit significant theoretical congruence with impression management. Within tourism settings, where visitors aspire to project civilized and cultivated self-images, these historical norms provide socially sanctioned behavioral protocols. Through comity behaviors, tourists effectively implement positive impression management strategies that align with societal expectations.

From the perspective of modern psychological theories, norm activation theory and impression management theory provide scientific theoretical foundations for studying and guiding comity behaviors in tourism photography. Norm activation theory elucidates how tourists develop and activate normative consciousness regarding comity behaviors across different situational contexts, enabling the implementation of effective interventions to promote such conduct. Impression management theory offers insights into how tourists strategically employ comity practices to cultivate positive self-images during photographic activities, thereby gaining social approval and recognition that reinforces continued civilized behavior. Therefore, this study's integration of traditional Chinese comity norms with contemporary psychological frameworks not only facilitates deeper exploration of the underlying mechanisms governing photographic comity behaviors but also establishes more compelling theoretical support and explanatory frameworks for empirical investigations. This interdisciplinary approach holds substantial significance for comprehensively understanding and effectively guiding tourists' civilized conduct in tourism contexts.

## Research hypothesis

### Norm activation and photo courtesy behavior

According to normative activation theory, the activation of social norms and individual norms is a prerequisite for the implementation of photographic courtesy. Social norms, which serve as standards for individual behavior in social interactions, encapsulate the expectations of social groups for individual conduct. In crowded tourism situations, tourists may not only be influenced by the evaluations and atmosphere of those around them but also by the relevant rules, norms, and reminders of the tourist destination, prompting polite behaviors when taking pictures.

Personal norms are self-imposed behavioral standards that individuals adhere to when performing certain actions and can better predict altruistic behavior (Schwartz, 1977). When personal norms are activated, individuals will act in accordance with them; otherwise, they may experience self-punishment through emotions such as guilt, regret, and shame (Li and Wang, 2019). Regarding the courtesy behavior of tourists, they are more likely to exhibit courteous behavior when they are more aware of the negative consequences of not taking photos politely and feel a stronger sense of personal responsibility.

When tourists' social and individual norms are activated, they may experience strong feelings of guilt and self-blame if they do not engage in photo courtesy behavior. This sense of moral obligation prompts them to exhibit courteous behavior when taking photos (Zhang and Wan, 2021). Based on this understanding, the following hypotheses are proposed:

H1: Social norms significantly positively affect photo courtesy behavior.
H2: Individual norms significantly positively affect photo courtesy behavior.

### The mediating role of impression management motivation

Social norms are codes of conduct that each member of society consciously adheres to based on social culture, and they are reinforced by the informal sanctions of social groups (Li and Wang, 2019). In public arenas, social norms are typically expressed through preemptive moral restraint and post-event criticism, which can trigger impression management motivation when individuals realize that their behavior is or will be evaluated by others (Morrison and Bies, 1991). Individual norms are individuals' perceptions of their own responsibilities and obligations. Specifically, in the context of photo-taking courtesy, this means that tourists consider courteous behavior while taking photos as their own code of conduct and moral obligation during travel. This strong self-restraint helps maintain their own image through this constraint (Li and Bai, 2018). Therefore, this paper proposes the following hypotheses:

H3: Social norms have a significant positive impact on impression management motivation.
H4: Individual norms have a significant positive impact on impression management motivation.

Impression management motivation is a social psychological and behavioral tendency to make a positive impression on others. Social context is a necessary condition for impression management, and the openness of the situation affects impression management (Li and Guo, 1999; Schlenker, 1980). The more open the situation, the more likely an individual is to engage in impression management and the easier it is to engage in altruistic behavior (Du et al., 2022). In the process of tourism, tourists with higher impression management motivation tend to have a higher tendency to take pictures courteously. It is evident that impression management motivation has a strong predictive effect on tourists' photo courtesy behavior, regardless of whether the behavior is intended to enhance their image or is a last resort measure to save face. Therefore, the following hypothesis is proposed:

H5: Impression management motivation has a significant positive impact on photo courtesy behavior.

In summary, this study selects impression management motivation as a mediating factor between normative activation and photo courtesy behavior. It examines whether impression management motivation mediates the relationship between social norms, individual norms, and photo courtesy behavior. The study also investigates if there is a significant difference in the mediating effect between the two paths: “social norms → impression management motivation → photo courtesy behavior” and “individual norms → impression management motivation → photo courtesy behavior.” Based on this, the following hypotheses are proposed:

H6a: Impression management motivation mediates the relationship between social norms and photo courtesy behavior.
H6b: Impression management motivation mediates the relationship between individual norms and photo courtesy behavior.

### The moderating role of proactive personality

Since social norms and individual norms affect impression management motivation, do individual personality traits have a moderating effect? To answer this question, this study introduces a variable closely related to the research object, namely proactive personality, and explores its possible moderating role in the process by which social norms and individual norms influence impression management motivation. Proactive personality refers to an individual's stable behavioral tendency to actively seek solutions without being constrained by the external environment (Bateman and Crant, 1993). As an important personality trait, proactive personality can help individuals take the initiative to exhibit courteous photo-taking behavior and demonstrate and lead in promoting civilized tourism. It can, therefore, have an impact on impression management motivation to a certain extent. Specifically, according to the investment principle of resource conservation theory, individuals with strong proactive personalities will make full use of positive psychological resources to gain benefits, further invest resources to avoid resource depletion, or recover from losses (Crant, 2000; Hobfoll, 1989). In public places, when social norms and individual norms are activated, tourists with strong proactive personalities are adept at mobilizing psychological resources to construct a positive understanding of social and individual norms, thereby stimulating impression management motivation. Conversely, tourists with weak proactive personalities tend to focus on their own needs and convenience, lacking active empathy. Thus, the impact of social norms and individual norms on impression management motivation is likely to be insignificant, weakening the effect on impression management motivation.

In summary, the following hypotheses are proposed:

H7a: Proactive personality positively moderates the relationship between social norms and impression management motivation.
H7b: Proactive personality positively moderates the relationship between individual norms and impression management motivation.

**Figure 1:**
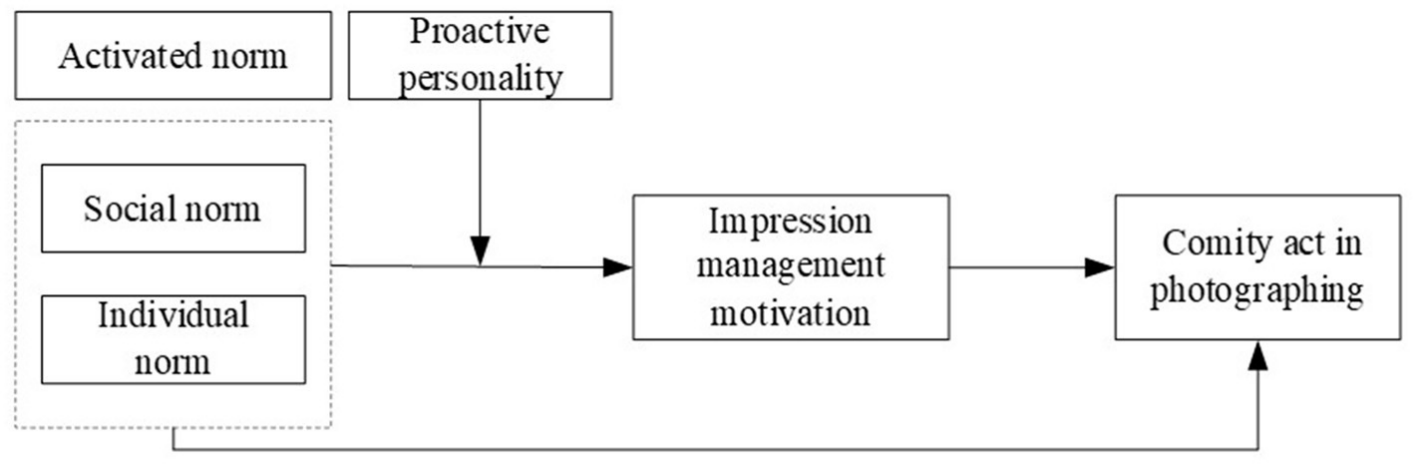
Theoretical research model.

In conclusion, a theoretical model was constructed in this study, as shown in Figure 1.

## Research design and data collection

### Questionnaire design

The questionnaire consists of three main parts. Firstly, respondents are provided with instructions ensuring anonymity and confidentiality of their responses, along with a brief explanation of the study's purpose. The main body of the questionnaire comprises five modules: social norms, individual norms, impression management motivation, photo courtesy behavior, and proactive personality. Each module is assessed using a Likert 7-point scale.

### Variable measurement

Drawing on authoritative literature and established scales from both domestic and international sources, this study involved two experts in tourism management and two experts in English to translate and back-translate foreign scales, adapting them to fit the Chinese context and ensuring the accuracy of measurement item expressions (Tu and Lin, 2021). The translation-back-translation process incorporated cultural adaptations to align items with Chinese tourism contexts. Western-culturally specific scenarios were replaced with authentic Chinese tourist experiences. A pilot survey with 100 respondents, representing a demographically diverse sample, validated the scale's readability and comprehension. Participant feedback helped refine ambiguous items, ensuring the measurement's validity and reliability for formal surveys. The final scale was determined after preliminary testing and corrections through a small sample survey in the initial stages. Except for control variables, all variables were measured using a Likert 7-point scale, where 1 signifies “strongly disagree” and 7 signifies “strongly agree.” Survey data were processed using SPSS 26.0 and AMOS 24.0 software.

The dependent variable in this study is photo courtesy behavior, encompassing six measurement items that assess tourists' choices in behaviors such as “actively giving way” and “quickly offering courtesy,” drawing on research scales by scholars like Hu (2009) and Li and Bai (2018). The independent variables include social norms and individual norms. Based on studies by Chen and Ma (2011) and Zhang et al. (2023), five measurement items were designed to gauge tourists' agreement with statements like “travel prompts remind me to behave politely when taking photos” and “my fellow travelers believe I should behave politely when taking photos.” Measurement items for individual norms were primarily derived from scales developed by Zhang and Wan (2021) and Zhou and Zhao (2023), with five items assessing tourists' perceptions such as “I believe it is appropriate for tourists to take photos” and “Influencing other tourists' photo-taking behavior makes me feel guilty.”

Impression management motivation serves as the mediating variable, with measurement items based on scales by McFarland et al. (2023) and Li and Bai (2018), encompassing four items assessing tourists' perceptions of “being recognized” and “concern for their image.”

The moderator variable, proactive personality, was measured using a scale developed by Seibert et al. (1999) and research by Qu et al. (2023), with six items such as “I am always looking for better ways to accomplish things” and “I actively seek to change my surroundings wherever I am.”

Control variables include tourists' personal characteristics, such as gender, age, education, monthly income, and annual trips.

### Data collection

For convenience, the research team conducted a preliminary survey of tourists in the Xi'an Datang Sleepless City Scenic Area, collecting a total of 145 valid samples. The test results showed that the Cronbach's α values for each construct scale were above 0.7, and the factor loadings for each item were above 0.7 (*p* < 0.05), indicating acceptable reliability and validity of the scale.

The data for this study were primarily collected by the research team between April and October 2023 from several locations, including the Xinjiang Tianshan Grand Canyon Scenic Area, Xi'an Datang Sleepless City Scenic Area, and Shanghai Disneyland. The survey targeted domestic tourists. Researchers distributed the questionnaires randomly at high-traffic areas such as scenic spot exits and rest areas. A total of 700 questionnaires were distributed, and 613 were returned, resulting in a recovery rate of 87.57%. After excluding questionnaires with identical answers for all items and incomplete responses, 599 valid questionnaires were retained, yielding an effective rate of 85.58%.

## Empirical analysis and results

### Descriptive statistical analysis of the sample

Among the 599 valid questionnaires, the demographic characteristics are as follows (Table 1): In terms of gender, the distribution is nearly equal, with women accounting for 56.4%. Regarding age, 25.4% of the respondents are under 30 years old, 22.4% are between 30 and 39 years old, 20.4% are between 40 and 49 years old, and 31.9% are 50 years old or above. The distribution of education levels shows that, except for postgraduate degrees (14.2%), the proportion of other educational qualifications ranges between 20% and 25%, indicating a balanced distribution. In terms of monthly income, 17.5% of respondents earn more than 20,001 yuan, while the proportions for other income levels are ~20%. Regarding the number of trips per year, 38.1% travel twice or less, 33.9% take 3 to 5 trips, and 28.0% travel six times or more. Overall, the demographic and tourism characteristics of the sample are well-represented, meeting the data requirements for subsequent empirical research.

**Table 1:** Sample descriptive statistical results.

**Demographic**	**Type**	**Frequency**	**Rate**
Gender	Female	338	56.4
	Male	261	43.6
Age	Under 30	152	25.4
	30 ~ 39	134	22.4
	40 ~ 49	122	20.4
	50 ~ 59	115	19.2
	Over 60	76	12.7
Education	Junior high school and below	136	22.7
	High school/Vocational school	136	22.7
	Associate degree	122	20.4
	Bachelor's degree	120	20.0
	Master's degree or above	85	14.2
Monthly income	< 5,000 yuan	118	19.7
	5,000 ~ 10,000 yuan	129	21.5
	10,001 ~ 15,000 yuan	131	21.9
	15,001~ 20,000 yuan	116	19.4
	20,001 and above	105	17.5
Annual number of trips	< 2 times	228	38.1
	3 ~ 5 times	203	33.9
	Over 6 times	168	28.0

### Common method bias test and confirmatory factor analysis

Despite employing a time interval design during data collection, common method bias might still be present. To address this, both the “Harman single-factor method” and the “control unmeasured single method latent factor method” were used to test for common method bias. First, the “Harman one-factor method” was applied, and the results showed that the unrotated first factor accounted for 39.19% of the variance, which is below the 50% threshold, indicating that common method bias is not a serious concern in this study. Secondly, adding a method latent factor to the benchmark model did not result in a significant change in the suitability index of the model, further confirming the absence of serious common method bias.

Confirmatory factor analysis was performed using Amos 24.0 (see Table 2). The fitting indices of the benchmark model were ideal (χ^2^ = 308.676, df = 289, χ^2^/df = 1.068, GFI = 0.963, TLI = 0.998, RMSEA = 0.011, SRMR = 0.025) and significantly better than those of other competing models. RMSEA (Root Mean Square Error of Approximation) is an indicator that measures model fit. A smaller RMSEA indicates a better fit, with values below 0.08 suggesting a good fit. GFI (Goodness of Fit Index) shows how well the model fits the data, with values closer to 1 indicating a better fit. TLI (Tucker-Lewis Index), an adjusted measure of fit, also indicates a good model fit when it is close to 1. SRMR (Standardized Root Mean Square Residual) reflects discrepancies between predicted and observed values. A smaller SRMR is preferred, with values under 0.08 being considered acceptable. In this study, all fit indices fall within the recommended thresholds, demonstrating a good model fit for the proposed framework.

**Table 2:** The results of confirmatory factor analysis.

**Model**	**χ^2^**	**df**	**χ^2^/df**	**CFI**	**TLI**	**RMSEA**	**Model comparison test**			
							**Model comparison**	Δχ^2^	Δ**df**	***P***
1. Reference model	308.676	289	1.068	0.998	0.998	0.011				
2. Four-factor model	1,997.914	293	6.819	0.867	0.852	0.099	2 vs.1	1,689.238	4	0.000
3. Three-factor model	3,152.866	296	10.652	0.777	0.755	0.127	2 vs.1	1,154.952	3	0.000
4. Two-factor model	4,473.404	298	15.011	0.673	0.644	0.153	2 vs.1	1,320.538	2	0.000
5. One-factor model	6,949.856	299	23.244	0.480	0.434	0.193	2 vs.1	2,476.452	1	0.000

### Scale reliability and validity test

First, Cronbach's α value was used to test the reliability of the scale. The results showed that the Cronbach's α values for the five factors were 0.925, 0.909, 0.876, 0.955, and 0.929, respectively, all exceeding the 0.7 threshold, indicating good reliability of the scale. The content of the measured items was derived from established scales, revised by experts, and pre-tested to ensure high content validity. Secondly, using Amos 24.0, a confirmatory factor model was established, and the measurement model's fit indices were as follows: χ^2^/df = 1.068, GFI = 0.963, AGFI = 0.955, CFI = 0.998, TLI = 0.998, IFI = 0.998, RMSEA = 0.011, and SRMR = 0.025. All these indicators met the standards proposed by Wu (2013).

The factor loadings for each item were higher than 0.7, the composite reliability (CR) for each variable exceeded 0.7, and the average variance extracted (AVE) value was higher than 0.5 (Table 3), indicating good convergent validity (Liu et al., 2017). The diagonal bold values in Table 4 represent the square roots of each variable's AVE, which are greater than the correlation coefficients between the corresponding variable and other variables, demonstrating good discriminant validity (Hair et al., 2010).

**Table 3:** The test of reliability and validity.

**Variable**	**Items**	**Non-standard loading**	**S.E**.	**Z value**	***P*-value**	**Factor loading**	**Cronbach'α**	**CR**	**AVE**
Social norm	SN1	1.000				0.759	0.925	0.928	0.721
	SN2	1.010	0.049	20.555	^***^	0.796			
	SN3	1.071	0.047	22.564	^***^	0.861			
	SN4	1.078	0.046	23.598	^***^	0.894			
	SN5	1.089	0.044	24.551	^***^	0.925			
Individual norm	IN1	1.000				0.752	0.909	0.912	0.675
	IN2	1.019	0.052	19.446	^***^	0.775			
	IN3	0.984	0.048	20.458	^***^	0.810			
	IN4	1.059	0.048	22.046	^***^	0.866			
	IN5	1.029	0.045	22.908	^***^	0.897			
Impression management motivation	IMM1	1.000				0.748	0.876	0.879	0.645
	IMM2	0.948	0.051	18.448	^***^	0.763			
	IMM3	1.030	0.051	20.354	^***^	0.838			
	IMM4	1.030	0.049	20.824	^***^	0.859			
Comity act in photographing	CAP1	1.000				0.793	0.955	0.956	0.785
	CAP2	1.096	0.044	24.859	^***^	0.865			
	CAP3	1.091	0.043	25.557	^***^	0.882			
	CAP4	1.128	0.042	26.723	^***^	0.910			
	CAP5	1.114	0.040	27.528	^***^	0.928			
	CAP6	1.117	0.040	27.653	^***^	0.931			
Proactive personality	PP1	1.000				0.762	0.929	0.931	0.693
	PP2	0.985	0.049	20.291	^***^	0.786			
	PP3	0.977	0.045	21.468	^***^	0.824			
	PP4	1.017	0.044	22.942	^***^	0.871			
	PP5	0.953	0.042	22.580	^***^	0.860			
	PP6	0.982	0.042	23.406	^***^	0.886			

^***^p < 0.001, ^**^p < 0.01, ^*^p < 0.05.

**Table 4:** The test of discriminant validity.

**Variable**	**M**	**SD**	**1**	**2**	**3**	**4**	**5**
1. Proactive personality	5.340	0.855	**0.832**				
2. Comity act in photographing	5.467	1.076	0.255	**0.886**			
3. Impression management motivation	4.727	0.921	0.226	0.684	**0.803**		
4. Individual norm	5.198	0.872	0.411	0.542	0.471	**0.822**	
5. Social norm	4.930	0.966	0.345	0.389	0.345	0.453	**0.849**

Square root of AVE is on the diagonal (in bold). Inter-construct correlations are on the off-diagonal.

## Hypothesis testing

### Path hypothesis testing

In this study, a hierarchical regression method was used to verify the relationship between norm activation and photo courtesy behavior, controlling for demographic characteristic variables. According to Table 5, there was a significant positive relationship between social norms and impression management motivation (β = 0.222, *p* < 0.001) as well as between individual norms and impression management motivation (β = 0.512, *p* < 0.001), indicating that both social norms and individual norms could stimulate impression management motivation. Additionally, the regression R^2^ in model 4 showed significant improvement compared to models 2 and 3, supporting the validation of H1 and H2.

**Table 5:** Results of regression analysis.

**Variables**	**Impression management motivation**				**Comity act in photographing**				
	**Model 1**	**Model 2**	**Model 3**	**Model 4**	**Model 5**	**Model 6**	**Model 7**	**Model 8**	**Model 9**
Gender	0.245^***^ (0.073)	0.265^***^ (0.066)	0.316^***^ (0.060)	0.317^***^ (0.058)	0.224^*^ (0.087)	0.251^**^ (0.078)	0.318^***^ (0.068)	0.046 (0.070)	0.197^**^ (0.063)
Age	0.103^***^ (0.026)	0.164^***^ (0.025)	0.189^***^ (0.022)	0.210^***^ (0.022)	0.111^***^ (0.032)	0.189^***^ (0.029)	0.224^***^ (0.025)	0.036 (0.025)	0.170^***^ (0.025)
Education	0.095^***^ (0.026)	0.139^***^ (0.024)	0.180^***^ (0.022)	0.192^***^ (0.022)	0.087^**^ (0.032)	0.144^***^ (0.029)	0.200^***^ (0.025)	0.018 (0.025)	0.141^***^ (0.024)
Monthly income	−0.001 (0.026)	0.007 (0.024)	0.005 (0.021)	0.009 (0.021)	0.000 (0.031)	0.010 (0.028)	0.009 (0.024)	0.001 (0.025)	0.009 (0.022)
Number of annual trips	0.234^***^ (0.045)	0.255^***^ (0.041)	0.292^***^ (0.037)	0.296^***^ (0.036)	0.144^**^ (0.053)	0.172^***^ (0.048)	0.221^***^ (0.042)	−0.027 (0.043)	0.111^**^ (0.040)
Social norm		0.391^***^ (0.035)		0.222^***^ (0.033)		0.504^***^ (0.041)			0.195^***^ (0.036)
Individual norm			0.605^***^ (0.036)	0.512^***^ (0.037)			0.797^***^ (0.041)		0.483^***^ (0.045)
Impression management motivation								0.729^***^ (0.039)	0.384^***^ (0.044)
constant	3.631^***^ (0.163)	1.346^***^ (0.254)	−0.148 (0.261)	−0.864^**^ (0.273)	4.549^***^ (0.195)	1.601^***^ (0.299)	−0.428 (0.296)	1.902^***^ (0.210)	−1.001^***^ (0.292)
Observations	599	599	599	599	599	599	599	599	599
R-squared	0.096	0.252	0.390	0.433	0.050	0.240	0.424	0.402	0.535
Adj R-squared	0.088	0.244	0.384	0.426	0.042	0.233	0.418	0.396	0.529
F	12.590	33.180	63.020	64.470	6.290	31.200	72.590	66.450	85.000

^***^ p < 0.001, ^**^ p < 0.01, ^*^ p < 0.05.

According to model 9 in Table 5, there was a significant positive relationship between social norms and photo courtesy behavior (β = 0.195, *p* < 0.001), individual norms and photo courtesy behavior (β = 0.483, *p* < 0.001), and impression management motivation and photo courtesy behavior (β = 0.384, *p* < 0.001). This indicates that social norms, individual norms as pre-variables, and impression management motivation as a mediating variable all had a significant positive impact on tourists' photo courtesy behavior. Moreover, the *R*^2^ of the regression equation in model 9 was significantly higher than that of other models, supporting the validation of H3, H4, and H5.

### Mediator effect test

Previous studies mostly refer to the step-by-step method (B-K) for mediating tests, supplemented by the Sobel test. The Bootstrap test has gradually replaced the B-K test and the Sobel test as a highly recognized mediator analysis method due to its higher validity and accuracy (Liu et al., 2017). In this study, the Bootstrap method was used to test the mediating effect of impression management motivation between normative stimuli and photo courtesy behavior. The Bootstrap sample size was set to 1000, the confidence level was set to 95%, and the mediation effect was considered significant if the 95% confidence interval of the indirect effect did not include 0 (Liu et al., 2017). The results are shown in Table 6.

**Table 6:** Mediation effect analysis results.

**Path relationship**	**Point estimate**	**Product of coefficients**		**Bootstrapping**	
				**BC 95% CI**	
		**SE**	**Z**	**Lower**	**Upper**
**Direct effect**					
EF1.SNORM → CAP	0.195	0.036	5.417	0.130	0.273
EF2.INORM → CAP	0.483	0.047	10.277	0.390	0.575
Total1	0.678	0.057	11.895	0.572	0.796
**Indirect effect**					
EF3.SNORM → IMM → CAP	0.085	0.015	5.667	0.057	0.117
EF4.INORM → IMM → CAP	0.197	0.025	7.880	0.152	0.247
Total2	0.282	0.033	8.545	0.220	0.352
**Total effect**					
Total1+Total2	0.960	0.052	18.462	0.864	1.068
**Effect comparison**					
EF2 VS. EF1	0.289	0.062	4.661	0.169	0.408
EF4 VS. EF3	0.111	0.025	4.440	0.068	0.163
**Effect ratio**					
EF3/Total	0.089	0.016	5.514	0.060	0.122
EF4/Total	0.205	0.027	7.675	0.155	0.259
Total1/Total	0.706	0.036	19.882	0.632	0.774
Total2/Total	0.294	0.036	8.260	0.226	0.368

Firstly, the direct effect of norm activation (social norms and individual norms) on photo courtesy behavior was significant. The direct effect of social norms on photo courtesy behavior was significant (β = 0.195), with a BC confidence interval of [0.130, 0.273], excluding 0. The direct effect of individual norms on photo courtesy behavior was also significant (β = 0.483), with a BC confidence interval of [0.390, 0.575], excluding 0. The sum of the direct effects of social norms and individual norms on photo courtesy behavior was 0.678, and the BC confidence interval did not contain 0, indicating a direct impact on photo courtesy behavior.

Among the indirect effects, the mediating effect of the path “social norms → impression management motivation → photo courtesy behavior” was significant (β = 0.085), with a BC confidence interval of [0.057, 0.117], excluding 0. The mediating effect of the path “individual norms → impression management motivation → photo courtesy behavior” was also significant (β = 0.197), with a BC confidence interval of [0.152, 0.247], excluding 0. These results indicate that impression management motivation plays a mediating role in the relationship between normative stimuli and photo courtesy behavior. The sum of the mediating effects of impression management motivation in the two paths was 0.282, with a BC confidence interval of [0.220, 0.352], excluding 0, supporting H6a and H6b.

Secondly, the difference between the direct effect of individual norms and social norms on photo courtesy behavior was 0.289, with a BC confidence interval of [0.169, 0.408], excluding 0. This indicates that the influence of individual norms on photo courtesy behavior is significantly higher than that of social norms. The difference between the mediating effects of impression management motivation in the two indirect paths was 0.111, with a BC confidence interval of [0.068, 0.163], excluding 0. This shows that the mediating role of impression management motivation differs significantly between the two paths, with the indirect influence of impression management motivation being greater in the “individual norms → impression management motivation → photo courtesy behavior” path.

Finally, in the theoretical model constructed in this study, the indirect effect value of normative stimuli on photo courtesy behavior through impression management motivation was 0.282, accounting for 29.4% of the total effect size. The BC confidence interval was [0.226, 0.368], excluding 0, indicating that the mediating effect of impression management motivation in the relationship between norm activation and photo courtesy behavior accounted for about 30%, with significant statistical explanatory power.

### Moderating effect test

**Figure 2:**
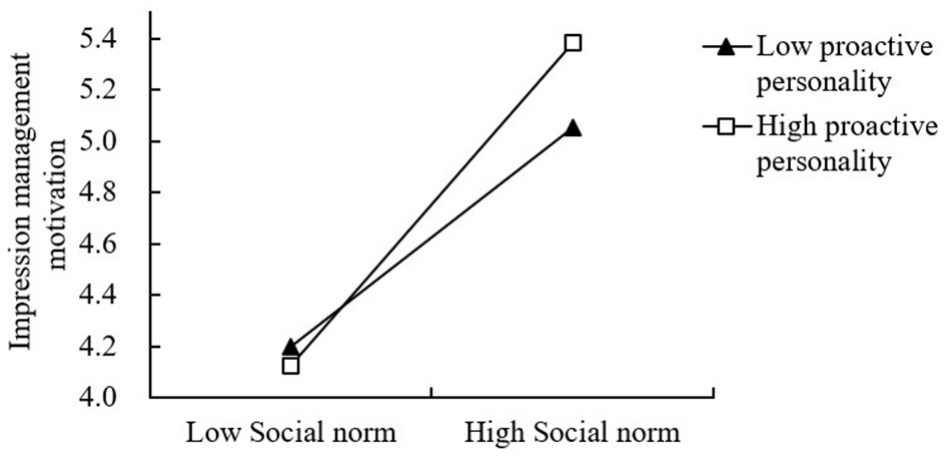
Moderating effect.

The moderating effect of active personality on the relationship between social norms and impression management motivation is shown in Model 4 in Table 7. The interaction terms between social norms and active personality had no significant effect on impression management motivation (β = 0.054, *P* > 0.05), indicating that the moderating effect of active personality on the relationship between social norms and impression management motivation is not significant. Therefore, hypothesis H7a is not supported. However, the results of Model 8 showed that the interaction terms between individual norms and active personality had a significant effect on impression management motivation (β = 0.136, *P* < 0.001), indicating that the moderating effect of active personality on the relationship between individual norms and impression management motivation is significant. Hence, hypothesis H7b is supported. To further clarify the moderating effect of proactive personality, this study plotted the moderating effect in the relationship between proactive personality and impression management motivation, as shown in Figure 2. Theoretically, the moderating effect of proactive personality manifests significance solely between individual norms and impression management motivation, while remaining non-significant in the context of social norms. This phenomenon may be attributed to core characteristics of proactive personality. Individuals with proactive tendencies demonstrate heightened propensity to actively shape environments and initiate goal-directed actions, prioritizing personal accountability and intrinsic values over mere social conformity. Individual norms, emphasizing personal obligations and duties, inherently align with the self-determined motivational orientation of proactive personalities. When individual norms prevail, proactive individuals more effectively translate internalized responsibility cognitions into behavioral motivations—such as impression management endeavors to sustain positive self-presentation. Conversely, social norms predominantly derive their influence from external pressures and collective expectations. Proactive individuals' characteristic independence may attenuate their sensitivity to social normative demands, predisposing them to act upon personal value systems rather than external conformity imperatives. Thus, proactive personality significantly moderates the individual norm-impression management pathway, whereas its moderating role proves non-significant within the social norm framework.

**Table 7:** Results of moderating effect test.

**Variables**	**Impression management motivation**							
	**Model 1**	**Model 2**	**Model 3**	**Model 4**	**Model 5**	**Model 6**	**Model 7**	**Model 8**
Gender	0.265^***^ (0.066)	0.264^***^ (0.070)	0.274^***^ (0.066)	0.277^***^ (0.066)	0.316^***^ (0.060)	0.264^***^ (0.070)	0.318^***^ (0.060)	0.310^***^ (0.059)
Age	0.164^***^ (0.025)	0.120^***^ (0.026)	0.167^***^ (0.024)	0.167^***^ (0.024)	0.189^***^ (0.022)	0.120^***^ (0.026)	0.190^***^ (0.022)	0.190^***^ (0.022)
Education	0.139^***^ (0.024)	0.117^***^ (0.026)	0.147^***^ (0.024)	0.146^***^ (0.024)	0.180^***^ (0.022)	0.117^***^ (0.026)	0.182^***^ (0.022)	0.184^***^ (0.022)
Monthly income	0.007 (0.024)	0.009 (0.025)	0.012 (0.024)	0.011 (0.024)	0.005 (0.021)	0.009 (0.025)	0.007 (0.021)	0.004 (0.021)
Number of annual trips	0.255^***^ (0.041)	0.234^***^ (0.043)	0.253^***^ (0.040)	0.255^***^ (0.040)	0.292^***^ (0.037)	0.234^***^ (0.043)	0.290^***^ (0.037)	0.304^***^ (0.037)
Social norm	0.391^***^ (0.035)		0.352^***^ (0.037)	0.120 (0.197)				
Proactive personality		0.262^***^ (0.041)	0.145^***^ (0.040)	−0.069 (0.184)		0.262^***^ (0.041)	0.057 (0.038)	−0.632^**^ (0.197)
Social norm × Proactive personality				0.045 (0.037)				
Individual norm					0.605^***^ (0.036)		0.585^***^ (0.038)	−0.121 (0.202)
								0.136^***^ (0.038)
Individual norm × Proactive personality								
Constant	1.346^***^ (0.254)	2.089^***^ (0.290)	0.719^*^ (0.306)	1.819 (0.968)	−0.148 (0.261)	2.089^***^ (0.290)	−0.354 (0.294)	3.157^**^ (1.029)
Observations	599	599	599	599	599	599	599	599
R-squared	0.252	0.153	0.268	0.270	0.390	0.153	0.392	0.405
Adj R-squared	0.244	0.145	0.259	0.260	0.384	0.145	0.385	0.397
F	33.180	17.830	30.870	27.210	63.020	17.830	54.470	50.180

^***^p < 0.001, ^**^p < 0.01, ^*^p < 0.05.

### Moderated mediating effect test

In this study, the SPSS macro program Process v4.2 was used to test the moderated mediating effect of active personality at three levels: low active personality (4.500, 16% quantile), medium active personality (5.333, 50% quantile), and high active personality (6.333, 84% quantile). Since the moderating effect of proactive personality on the relationship between social norms and impression management motivation is not significant, this section only reports the moderating effect of individual norms on the mediating variables of proactive personality in the path to photo courtesy behavior, as shown in Table 8. In the influence path of individual norms on photo courtesy behavior, the mediating effects of impression management motivation were 0.056, 0.088, and 0.126 at the low, medium, and high levels of active personality, respectively. The 95% Percentile and Bias-corrected confidence intervals did not include 0, indicating significant mediating effects. These results show that the higher the level of active personality, the stronger the mediating effect of impression management motivation. Therefore, there is a moderated mediating effect. Additionally, there was a significant difference between the 95% confidence intervals of the mediating effect differences of impression management motivation at low, medium, and high levels of active personality. This further indicates that active personality enhances the mediating effect between individual norms and photo courtesy behavior.

**Table 8:** Bootstrap test results of mediation moderated.

**Independent variable**	**Result type**	**Moderator**	**Effect size**	**Boot SE**	**Bootstrap 95% CI**			
					**Percentile**		**Bias-corrected**	
Individual norm	Moderated mediating effect	Low proactive personality	0.056	0.016	0.023	0.086	0.025	0.091
		Medium proactive personality	0.088	0.015	0.061	0.119	0.062	0.123
		High proactive personality	0.126	0.023	0.085	0.174	0.085	0.174
	Comparison of mediating effects	Medium-Low	0.032	0.012	0.011	0.056	0.012	0.056
		High-Low	0.07	0.025	0.025	0.123	0.026	0.124
		High-Medium	0.038	0.014	0.014	0.067	0.014	0.068

## Conclusion and discussion

### Research conclusions and theoretical contributions

Courtesy is a core value of Chinese culture that embodies respect, harmony, and wisdom. It requires individuals to show humility, self-discipline, and a pursuit of common interests in social interactions. Taking photos is a common behavior among tourists, and photo courtesy not only demonstrates an individual's civilized quality and respect for others but also reflects the value orientation of social harmony and collective interests. Based on norm activation theory, impression management theory, courtesy, and other related theories, this study focuses on tourists in tourism contexts. It constructs an action mechanism model with social norms and individual norms as independent variables, impression management motivation as the mediating variable, photo courtesy behavior as the outcome variable, and proactive personality as the moderating variable. The research data were obtained through both online and offline methods, and empirical tests were conducted. The following research conclusions were drawn: First, social norms and individual norms have a significant positive impact on photo courtesy behavior, with the direct impact of individual norms being significantly greater than that of social norms. Second, impression management motivation serves as an important mediating variable between norm activation and photo courtesy behavior, transmitting part of the effect of norm activation on photo courtesy behavior. The indirect effect of impression management motivation in the path of “individual norms → impression management motivation → photo courtesy behavior” is greater than the indirect effect in the path of “social norms → impression management motivation → photo courtesy behavior.” Third, proactive personality has a moderating effect between individual norms and impression management motivation, while the moderating effect between social norms and impression management motivation is not significant. Therefore, in the formation mechanism of tourists' photo courtesy behavior, the mediating effect of impression management motivation in the path of “individual norms → impression management motivation → photo courtesy behavior” varies depending on proactive personality. Specifically, in the group with high proactive personality, impression management motivation has a higher explanatory power in the relationship between individual norms and photo courtesy behavior.

This study contributes to the theoretical landscape by integrating Norm Activation Theory, Impression Management Theory, and the traditional Chinese culture of comity through an interdisciplinary lens. It constructs a comprehensive theoretical framework, offering a novel perspective for examining tourists' comity behaviors within the context of tourism photography. This theoretical amalgamation not only broadens the application horizons of existing theories but also provides the tourism behavior research field with more extensive and profound theoretical grounding.

The study extends **Norm Activation Theory**, which has traditionally focused on the influence of social norms on individual behavior. Here, it further explores the role of individual norms in tourists' comity behaviors during photography. Individual norms, defined as behavioral standards shaped by personal values and ethical benchmarks, are pivotal in the decision-making process. Empirical findings indicate that individual norms have a significantly greater direct effect on tourists' courteous photography behaviors than social norms. This discovery not only diversifies the scenarios where Norm Activation Theory applies but also offers a fresh angle for interpreting how individuals navigate social pressures and personal convictions in their behavioral choices.

**Impression Management Theory** is incorporated to investigate the role of impression management motivation in the interplay between social and individual norms and tourists' behaviors. Impression Management Theory examines how individuals shape others' perceptions and behaviors through information control. In travel photography, tourists' impression management motivations may drive them to exhibit comity to maintain a favorable image. This study underscores the often-overlooked yet crucial role of impression management motivation in the influence of social and individual norms on tourist behavior.

The study also integrates **the traditional Chinese culture of comity** into its theoretical framework. The culture of comity, emphasizing humility, self-restraint, and the pursuit of communal welfare, is a cornerstone of traditional Chinese culture. By intertwining this cultural aspect with modern tourism behavior studies, the research reflects the enduring impact of traditional Chinese culture on contemporary social conduct and provides fresh theoretical insights for cross-cultural tourism behavior research.

The theoretical synthesis presented in this study offers a new analytical scaffold for future research. The integration of diverse theories not only extends the reach of each individual theory but also enriches the tourism behavior research field with more holistic and profound theoretical support. This synthesis encourages the cross-pollination and convergence of theories from various disciplines within tourism studies, providing a more nuanced lens for interpreting tourist behaviors and a more efficacious roadmap for tourism management practices. In conclusion, the study's theoretical contributions are evident in the deepened and innovative synthesis of extant theories. It offers new perspectives and analytical tools for tourism behavior research, fostering a deeper academic understanding of the underlying mechanisms of tourist behavior. The expanded and integrated theoretical framework supplies new theoretical resources and research avenues for the field of tourism behavior studies.

## Practical implications

This study focuses on the civilized and courteous behavior of tourists in the context of taking pictures, deeply discussing the influence mechanism of normative activation on tourists' photo courtesy behavior. The research conclusions have important guiding significance for standardizing tourists' behavior and improving tourism management practice.

From the perspective of tourists, it can guide tourists to practice civilized and courteous behavior during travel. By improving self-regulation and leveraging active personality traits, tourists can internalize civilized and courteous behaviors into personal habits. It is recommended that tourists actively participate in tourism civilized behavior training and volunteer service activities, such as the “Civilized Tourism Ambassador” program. This helps tourists understand civilized tourism etiquette, improve personal quality, and spread the concept of civilized tourism in practice. Additionally, tourists can use social media and other platforms to share their experiences of civilized tourism, forming a positive demonstration effect. They should actively abide by the rules of courtesy when taking photos during travel, respect others, and show a good image as social citizens. Through these concrete actions, tourists can enhance their personal travel experience and contribute to the advancement of civilization in the tourism industry as a whole.

From the perspective of tourism enterprises, companies should take comprehensive measures to meet tourists' needs in scenic areas for taking pictures. First, enterprises should strengthen tourists' awareness of photo courtesy through the reasonable allocation of human resources, such as arranging professional service personnel for on-site management in popular scenic spots and setting up clear reminder signs, thus creating a civilized and harmonious tourism environment. Second, enterprises should optimize the spatial layout of scenic spots by designing multi-angle viewing platforms and photo spots, dispersing tourist flow, reducing waiting times, and encouraging photo-taking in different locations. Additionally, companies can use virtual reality (VR) technology to create virtual photo experience areas, providing visitors with unique photo experiences while reducing physical crowding. This guides tourists to plan their photo times reasonably and avoid peak hours. Finally, enterprises should promote the positive roles of social norms, individual norms, and impression management motives in encouraging photo courtesy behavior. By strengthening publicity and education on civilized tourism through media and social platforms, tourists' awareness and behavior of civilized courtesy can be stimulated. These strategies not only improve tourist satisfaction but also contribute to the advancement of civilization in the tourism industry.

From the perspective of industry management departments, positive guidance and psychological construction of tourists' social norms and individual norms should be strengthened to create a civilized travel atmosphere. First, the cultural and tourism industry management departments should formulate a detailed code of conduct for civilized tourism to provide clear references for such behavior. Second, they should carry out various publicity activities using social media, tourism festivals, and other platforms to improve public understanding of civilized tourism. Third, tourism management departments, tourism associations, and other organizations can cooperate with tourism enterprises to develop educational tools and materials, such as guides and promotional videos, to enhance tourists' awareness of civilized courtesy through multi-channel dissemination. Finally, establishing incentive mechanisms for practicing civilized and courteous tourism can motivate tourists. Providing scenic discounts, souvenirs, and other incentives can stimulate tourists' enthusiasm for civilized and courteous tourism, fostering a positive tourism culture.

## Research limitations and prospects

Although this study expands the explanatory perspective of the direct relationship between normative activation and tourists' photo courtesy behavior in the tourism context, there are still some limitations. First, this study follows previous research designs and adopts a cross-regional and cross-time questionnaire survey, which cannot completely avoid the possible risk of alternative interpretation. Future studies can adopt a blended study design, combining experimental methods and experiential sampling methods, and repeat the measurement of the main variables to better explore the development trend and causal relationship between the variables. Second, in terms of analysis techniques, this study uses class regression analysis to test the mediating effect of impression management motivation and the moderating effect of proactive personality. This approach may accumulate and amplify the error of model testing. To control the test error of the overall model more effectively, future research can consider using Mplus software to test the latent variable at one time with a mediating effect model of the moderated latent variable. Third, the samples in this study are from the Tianshan Grand Canyon Scenic Area in Xinjiang, the Tangbuye City Scenic Area in Xi'an, and Shanghai Disneyland. This does not include other types of scenic spots and attractions, which may lead to some differences in the impact of normative activation on impression management motivation and photo courtesy behavior. This study adopts a convenience sampling method, with samples limited to the aforementioned attractions, potentially introducing selection bias. Tourists at these attractions may exhibit homogeneity in terms of geographical origin, age, and consumption capacity, which may fail to adequately represent broader tourist populations and affect the generalizability of the conclusions. For example, the Xinjiang Tianshan Grand Canyon primarily attracts nature enthusiasts, the Xi'an Great Tang All-Day Mall tends to draw cultural history enthusiasts, while Shanghai Disneyland mainly caters to family tourists and young adults. Differences in yielding behavior during photography and related influencing factors among visitors at these distinct attractions may limit the research findings. Accordingly, future studies should expand sample sizes to include other types of attractions and conduct multi-group comparative analyses to verify the generalizability of the conclusions drawn in this study. Fourth, this study empirically analyzes the mechanism of normative activation on tourists' photo courtesy behavior and verifies the mediating effect of impression management motivation without considering other mediating variables. Future scholars can consider including other mediating variables when studying the influence of normative activation on photo courtesy behavior to improve the explanatory power between normative activation and photo courtesy behavior. Furthermore, future research should further explore the influence of emotional factors on tourists' comity behaviors in photography. For instance, tourists may experience frustration due to obstructed photography attempts or excitement from successful photo captures, both of which could significantly impact their subsequent photographic behaviors and willingness to yield. Studies could incorporate variables such as emotion regulation strategies to examine their potential mediating or moderating roles between emotional states and photographic conduct, thereby providing richer perspectives for understanding and guiding civilized photography practices. Concurrently, with the advancement of digital media, tourists' perceptions of social norms and approaches to impression management are undergoing transformations. Social interactions, photo-sharing practices, and feedback mechanisms on digital platforms may reshape tourists' understanding of normative expectations and their prioritization of impression management. Future investigations could focus on characterizing photographic behaviors in digital media environments and analyzing how these platforms reconfigure tourists' social norm cognition and impression management motivations. Such explorations would offer more contemporarily relevant insights for tourism management and marketing strategies.

## Data Availability

The raw data supporting the conclusions of this article will be made available by the authors, without undue reservation.
